# Crystal structure of ethyl 2-{2-[(1*Z*)-1-hy­droxy-3-(4-nitro­phen­yl)-3-oxoprop-1-en-1-yl]phen­oxy}acetate

**DOI:** 10.1107/S2056989015020794

**Published:** 2015-11-07

**Authors:** Shaaban K. Mohamed, Joel T. Mague, Mehmet Akkurt, Eman A. Ahmed, Mustafa R. Albayati

**Affiliations:** a Chemistry and Environmental Division, Manchester Metropolitan University, Manchester M1 5GD, England; b Chemistry Department, Faculty of Science, Minia University, 61519 El-Minia, Egypt; c Department of Chemistry, Tulane University, New Orleans, LA 70118, USA; d Department of Physics, Faculty of Sciences, Erciyes University, 38039 Kayseri, Turkey; e Department of Chemistry, Faculty of Science, Sohag University, 82524 Sohag, Egypt; f Kirkuk University, College of Science, Department of Chemistry, Kirkuk, Iraq

**Keywords:** crystal structure, aryl­oxyphen­oxy compounds, herbicides

## Abstract

The title compound, C_19_H_17_NO_7_, crystallized in a ratio of about 6:4 of the two possible keto–enol forms. This was observed as disorder over the central C_3_H_2_O_2_ unit. The dihedral angle between the rings is 8.2 (2)°.The mol­ecules pack by C—H⋯O interactions in a layered fashion parallel to (-104).

## Related literature   

For the use of aryl­oxyphen­oxy compounds in various herbicidal applications, see: Zhu *et al.* (2006 ▸, 2009 ▸); Li (2004 ▸); Wang *et al.* (2004 ▸). For the synthesis of the title compund, see: Akkurt *et al.* (2015 ▸).
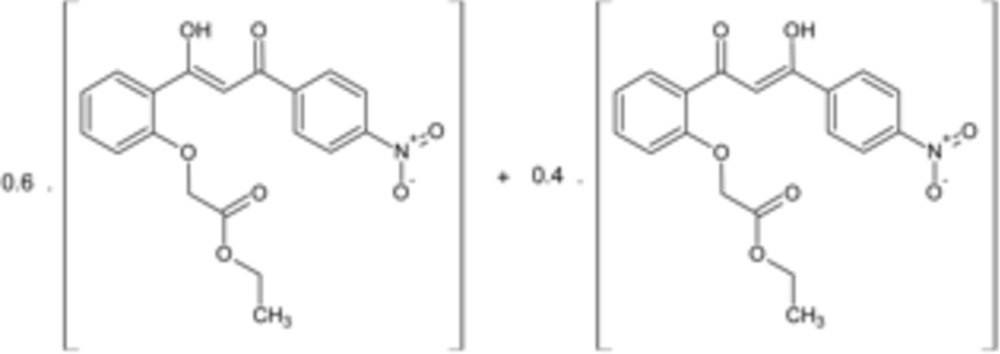


## Experimental   

### Crystal data  

C_19_H_17_NO_7_
*M* *_r_* = 371.33
Monoclinic, 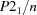

*a* = 4.7818 (10) Å
*b* = 16.260 (3) Å
*c* = 21.948 (5) Å
β = 95.933 (3)°
*V* = 1697.4 (6) Å^3^
*Z* = 4
Mo *K*α radiation
μ = 0.11 mm^−1^
*T* = 150 K
0.24 × 0.08 × 0.03 mm

### Data collection  

Bruker SMART APEX CCD diffractometer
Absorption correction: multi-scan (*SADABS*; Bruker, 2014 ▸) *T*_min_ = 0.60, *T*_max_ = 1.00
15039 measured reflections
3952 independent reflections
1800 reflections with *I* > 2σ(*I*)
*R*_int_ = 0.116

### Refinement  

*R*[*F* ^2^ > 2σ(*F* ^2^)] = 0.072
*wR*(*F* ^2^) = 0.194
*S* = 1.00
3952 reflections
245 parameters
H-atom parameters constrained
Δρ_max_ = 0.23 e Å^−3^
Δρ_min_ = −0.29 e Å^−3^

### 

Data collection: *APEX2* (Bruker, 2014 ▸); cell refinement: *SAINT* (Bruker, 2014 ▸); data reduction: *SAINT*; program(s) used to solve structure: *SHELXT* (Sheldrick, 2015*a* ▸); program(s) used to refine structure: *SHELXL2014* (Sheldrick, 2015*b* ▸); molecular graphics: *DIAMOND* (Brandenburg & Putz, 2012 ▸); software used to prepare material for publication: *SHELXTL* (Sheldrick, 2008 ▸).

## Supplementary Material

Crystal structure: contains datablock(s) global, I. DOI: 10.1107/S2056989015020794/qm2113sup1.cif

Structure factors: contains datablock(s) I. DOI: 10.1107/S2056989015020794/qm2113Isup2.hkl

Supporting information file. DOI: 10.1107/S2056989015020794/qm2113Isup3.cml

**Fig. 1.**
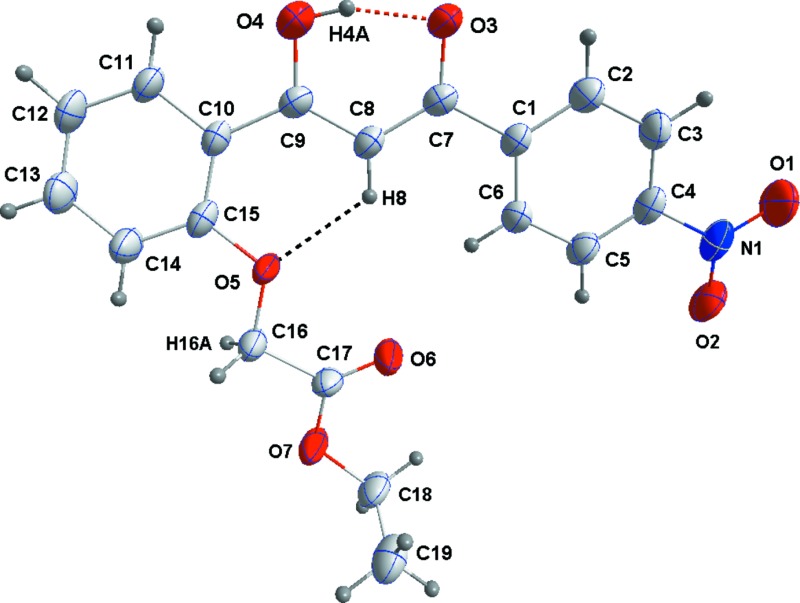
The title molecule with labeling scheme and 50% probability ellipsoids. Only one location (H4A) of the disordered enol hydrogen is shown. Intramolecular hydrogen bonds are shown by dotted lines.

**Fig. 2.**
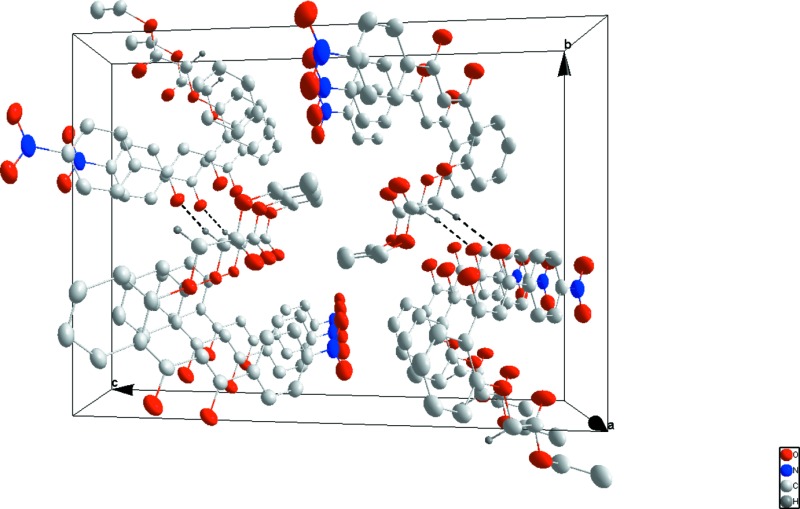
Packing viewed down the a axis. Intermolecular C—H···O hydrogen bonds are shown by dotted lines.

**Fig. 3.**
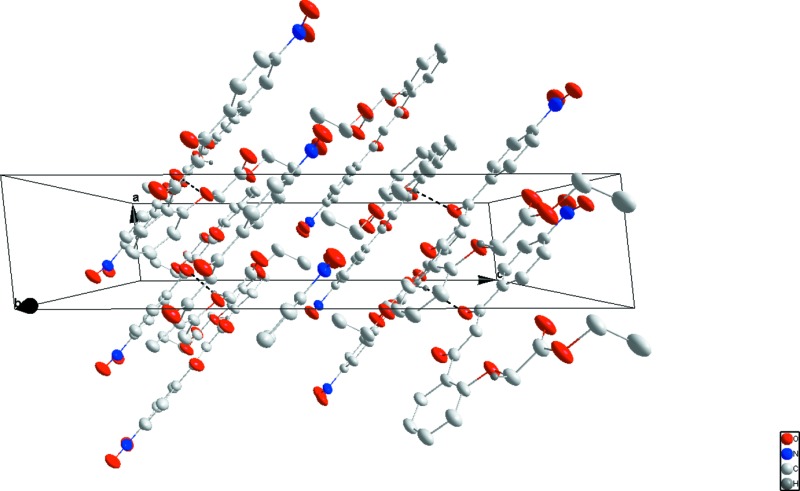
Packing viewed down the b axis showing the layered structure.

A . DOI: 10.1107/S2056989015020794/qm2113fig1.tif
The title mol­ecule with labeling scheme and 50% probability ellipsoids. Only one location (H4*A*) of the disordered enol hydrogen is shown. Intra­molecular hydrogen bonds are shown by dotted lines.

a . DOI: 10.1107/S2056989015020794/qm2113fig2.tif
Packing viewed down the *a* axis. Inter­molecular C—H⋯O hydrogen bonds are shown by dotted lines.

b . DOI: 10.1107/S2056989015020794/qm2113fig3.tif
Packing viewed down the *b* axis showing the layered structure.

CCDC reference: 1434730

Additional supporting information:  crystallographic information; 3D view; checkCIF report

## Figures and Tables

**Table 1:** Hydrogen-bond geometry (Å, °)

*D*—H⋯*A*	*D*—H	H⋯*A*	*D*⋯*A*	*D*—H⋯*A*
C8—H8⋯O5	0.95	2.18	2.796 (4)	122
C16—H16*A*⋯O3^i^	0.99	2.35	3.295 (5)	160
O3—H3*A*⋯O4	0.86	1.69	2.435 (3)	144
O4—H4*A*⋯O3	0.86	1.62	2.435 (3)	158

Symmetry code: (i) 
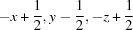
.

## supplementary crystallographic information

### S1. Comment

Aryloxyphenoxy propionates are an important class of herbicides due to their high efficiency, broad spectrum, low toxicity and good selectivity (Zhu *et al.*, 2006; Zhu *et al.*, 2009). Thus, aryloxy-phenoxy propionate herbicides such as fluazifop-butyl, heloxyfop-methyl, quizalofop-ethyl and cyhalofop-butyl have been developed (Li, 2004), and are widely used to control gramineous weeds. In addition, some aryloxy-phenoxy acetates exhibit good herbicidal activity. For example, two substituted pyrazolo[3,4-*d*] pyrimidin-4-yloxy phenoxy acetates display considerable activities (Wang *et al.*, 2004), with 100% inhibition against the root growth of Brassica napus *L*. Based on such facts, we report in this study the synthesis and crystal structural determination of the title compound.

In the title molecule, the dihedral angle between the C1–C6 ring and the mean plane of the central O3, C7, C8, C9, O4 unit is 2.8 (2)° while that between this latter plane and the C10–C15 ring is 8.2 (2)°. The molecule crystallized as a mixture of the two possible keto enol forms. This was observed as disorder over the central C_3_H_2_O_2_ unit. The molecules pack in a layered fashion (Figs. 2 and 3).

### S2. Experimental

The title compund was prepared according to our reported method (Akkurt *et al.*, 2015). Suitable crystals were obtained by slow evaporation method of a solution of the title compund in ethanol.

### S3. Refinement

H-atoms attached to carbon were placed in calculated positions (C—H = 0.95 - 0.99 Å). All were included as riding contributions with isotropic displacement parameters 1.2 - 1.5 times those of the attached atoms. From the equivalence of the C7—C8 and C8—C9 bond distances, the near equivalence of the C7—O3 and C9—O4 bond distances and the observance of only one peak attributable to a hydrogen attached to C8 in a difference map, it was concluded that the compound exists as the keto-enol tautomer with the enol hydrogen disordered between O3 and O4. Contoured difference maps calculated in the region between O3 and O4 showed an elongated region of density consistent with this assumption. The two components of the disordered hydrogen (H3a and H4a) were placed in positions consistent with forming intramolecular O—H···O hydrogen bonds and allowed to ride on the respective oxygen atoms.

### Crystal data

**Table:**

C_19_H_17_NO_7_	*F*(000) = 776
*M**_r_* = 371.33	*D*_x_ = 1.453 Mg m^−^^3^
Monoclinic, *P*2_1_/*n*	Mo *K*α radiation, λ = 0.71073 Å
*a* = 4.7818 (10) Å	Cell parameters from 2496 reflections
*b* = 16.260 (3) Å	θ = 2.3–26.7°
*c* = 21.948 (5) Å	µ = 0.11 mm^−^^1^
β = 95.933 (3)°	*T* = 150 K
*V* = 1697.4 (6) Å^3^	Column, pale yellow
*Z* = 4	0.24 × 0.08 × 0.03 mm

### Data collection

**Table:**

Bruker SMART APEX CCD diffractometer	3952 independent reflections
Radiation source: fine-focus sealed tube	1800 reflections with *I* > 2σ(*I*)
Graphite monochromator	*R*_int_ = 0.116
Detector resolution: 8.3660 pixels mm^-1^	θ_max_ = 27.9°, θ_min_ = 1.6°
φ and ω scans	*h* = −6→6
Absorption correction: multi-scan (*SADABS*; Bruker, 2014)	*k* = −20→20
*T*_min_ = 0.60, *T*_max_ = 1.00	*l* = −28→28
15039 measured reflections	

### Refinement

**Table:**

Refinement on *F*^2^	Primary atom site location: structure-invariant direct methods
Least-squares matrix: full	Secondary atom site location: difference Fourier map
*R*[*F*^2^ > 2σ(*F*^2^)] = 0.072	Hydrogen site location: mixed
*wR*(*F*^2^) = 0.194	H-atom parameters constrained
*S* = 1.00	*w* = 1/[σ^2^(*F*_o_^2^) + (0.0701*P*)^2^ + 0.4312*P*] where *P* = (*F*_o_^2^ + 2*F*_c_^2^)/3
3952 reflections	(Δ/σ)_max_ < 0.001
245 parameters	Δρ_max_ = 0.23 e Å^−^^3^
0 restraints	Δρ_min_ = −0.29 e Å^−^^3^

### Special details

**Table:**

Experimental. The diffraction data were collected in three sets of 363 frames (0.5° width in ω) at φ = 0, 120 and 240°. A scan time of 120 sec/frame was used.
Geometry. All e.s.d.'s (except the e.s.d. in the dihedral angle between two l.s. planes) are estimated using the full covariance matrix. The cell e.s.d.'s are taken into account individually in the estimation of e.s.d.'s in distances, angles and torsion angles; correlations between e.s.d.'s in cell parameters are only used when they are defined by crystal symmetry. An approximate (isotropic) treatment of cell e.s.d.'s is used for estimating e.s.d.'s involving l.s. planes.
Refinement. Refinement of *F*^2^ against ALL reflections. The weighted *R*-factor *wR* and goodness of fit *S* are based on *F*^2^, conventional *R*-factors *R* are based on *F*, with *F* set to zero for negative *F*^2^. The threshold expression of *F*^2^ > σ(*F*^2^) is used only for calculating *R*-factors(gt) *etc*. and is not relevant to the choice of reflections for refinement. *R*-factors based on *F*^2^ are statistically about twice as large as those based on *F*, and *R*- factors based on ALL data will be even larger. H-atoms attached to carbon were placed in calculated positions (C—H = 0.95 - 0.99 Å). All were included as riding contributions with isotropic displacement parameters 1.2 - 1.5 times those of the attached atoms. From the equivalence of the C7—C8 and C8—C9 bond distances, the near equivalence of the C7—O3 and C9—O4 bond distances and the observance of only one peak attributable to a hydrogen attached to C8 in a difference map, it was concluded that the compound exists as the keto-enol tautomer with the enol hydrogen disordered between O3 and O4. Contoured difference maps calculated in the region between O3 and O4 showed an elongated region of density consistent with this assumption. The two components of the disordered hydrogen (H3a and H4a) were placed in positions consistent with forming intramolecular O—H···O hydrogen bonds and allowed to ride on the respective oxygen atoms.

### Fractional atomic coordinates and isotropic or equivalent isotropic displacement parameters (Å^2^)

**Table:**

	*x*	*y*	*z*	*U*_iso_*/*U*_eq_	Occ. (<1)
O1	1.3713 (6)	0.88831 (19)	0.52969 (13)	0.0660 (9)	
O2	1.3341 (6)	0.75575 (19)	0.52420 (12)	0.0568 (8)	
O3	0.2970 (5)	0.93367 (15)	0.30167 (11)	0.0469 (7)	
H3A	0.1539	0.9368	0.2746	0.070*	0.4
O4	−0.0854 (5)	0.88128 (16)	0.23034 (12)	0.0559 (8)	
H4A	0.0513	0.9093	0.2485	0.084*	0.6
O5	−0.0494 (5)	0.63587 (13)	0.28606 (10)	0.0391 (6)	
O6	0.3347 (6)	0.58820 (15)	0.37708 (13)	0.0575 (8)	
O7	0.1989 (6)	0.45723 (15)	0.36325 (12)	0.0539 (8)	
N1	1.2619 (7)	0.8252 (2)	0.50788 (14)	0.0482 (9)	
C1	0.5850 (7)	0.8492 (2)	0.36892 (15)	0.0324 (8)	
C2	0.7260 (7)	0.9186 (2)	0.39276 (17)	0.0412 (9)	
H2	0.6703	0.9715	0.3775	0.049*	
C3	0.9464 (8)	0.9116 (2)	0.43836 (17)	0.0451 (10)	
H3	1.0410	0.9591	0.4551	0.054*	
C4	1.0263 (7)	0.8345 (2)	0.45906 (15)	0.0368 (9)	
C5	0.8938 (7)	0.7638 (2)	0.43615 (16)	0.0398 (9)	
H5	0.9535	0.7112	0.4510	0.048*	
C6	0.6703 (7)	0.7720 (2)	0.39061 (15)	0.0372 (9)	
H6	0.5753	0.7244	0.3742	0.045*	
C7	0.3471 (7)	0.8596 (2)	0.31935 (15)	0.0321 (8)	
C8	0.1895 (7)	0.7929 (2)	0.29482 (15)	0.0329 (8)	
H8	0.2333	0.7388	0.3091	0.039*	
C9	−0.0329 (7)	0.8058 (2)	0.24920 (15)	0.0331 (8)	
C10	−0.2205 (7)	0.7428 (2)	0.21933 (15)	0.0327 (8)	
C11	−0.4082 (7)	0.7682 (2)	0.16947 (15)	0.0384 (9)	
H11	−0.4063	0.8241	0.1568	0.046*	
C12	−0.5941 (7)	0.7148 (2)	0.13854 (16)	0.0440 (10)	
H12	−0.7185	0.7339	0.1049	0.053*	
C13	−0.6003 (8)	0.6340 (2)	0.15610 (16)	0.0448 (10)	
H13	−0.7287	0.5969	0.1347	0.054*	
C14	−0.4184 (8)	0.6064 (2)	0.20538 (16)	0.0437 (10)	
H14	−0.4233	0.5505	0.2177	0.052*	
C15	−0.2297 (7)	0.6604 (2)	0.23651 (15)	0.0353 (8)	
C16	−0.0496 (8)	0.5517 (2)	0.30174 (16)	0.0397 (9)	
H16A	−0.0199	0.5176	0.2656	0.048*	
H16B	−0.2324	0.5363	0.3160	0.048*	
C17	0.1850 (8)	0.5374 (2)	0.35214 (16)	0.0384 (9)	
C18	0.4091 (8)	0.4278 (2)	0.41157 (17)	0.0489 (10)	
H18A	0.5634	0.4683	0.4186	0.059*	
H18B	0.4899	0.3751	0.3990	0.059*	
C19	0.2724 (9)	0.4159 (3)	0.46874 (18)	0.0589 (12)	
H19A	0.1949	0.4683	0.4812	0.088*	
H19B	0.4117	0.3959	0.5013	0.088*	
H19C	0.1205	0.3755	0.4615	0.088*	

### Atomic displacement parameters (Å^2^)

**Table:**

	*U*^11^	*U*^22^	*U*^33^	*U*^12^	*U*^13^	*U*^23^
O1	0.0585 (19)	0.076 (2)	0.0572 (19)	−0.0132 (17)	−0.0234 (15)	−0.0046 (17)
O2	0.0511 (18)	0.073 (2)	0.0420 (16)	0.0133 (15)	−0.0162 (13)	0.0053 (15)
O3	0.0465 (15)	0.0384 (15)	0.0514 (17)	−0.0023 (12)	−0.0164 (13)	0.0115 (12)
O4	0.0570 (18)	0.0470 (17)	0.0582 (18)	−0.0019 (14)	−0.0211 (14)	0.0137 (14)
O5	0.0443 (15)	0.0320 (14)	0.0364 (14)	0.0022 (11)	−0.0179 (12)	0.0032 (11)
O6	0.0611 (18)	0.0403 (16)	0.0629 (19)	−0.0073 (14)	−0.0321 (15)	0.0070 (14)
O7	0.0628 (18)	0.0381 (16)	0.0534 (17)	0.0016 (13)	−0.0295 (14)	0.0066 (13)
N1	0.0387 (19)	0.073 (3)	0.0314 (18)	−0.0048 (19)	−0.0042 (15)	−0.0007 (18)
C1	0.0299 (18)	0.037 (2)	0.0298 (18)	0.0020 (16)	−0.0013 (15)	0.0016 (16)
C2	0.039 (2)	0.041 (2)	0.042 (2)	0.0028 (17)	−0.0036 (18)	0.0011 (17)
C3	0.044 (2)	0.047 (3)	0.042 (2)	−0.0021 (19)	−0.0090 (18)	−0.0069 (19)
C4	0.0300 (19)	0.053 (2)	0.0271 (19)	0.0017 (17)	0.0007 (15)	−0.0004 (17)
C5	0.036 (2)	0.045 (2)	0.037 (2)	0.0052 (17)	−0.0063 (17)	0.0045 (18)
C6	0.037 (2)	0.039 (2)	0.035 (2)	−0.0024 (16)	−0.0027 (16)	0.0030 (17)
C7	0.0322 (19)	0.034 (2)	0.0304 (19)	0.0040 (16)	0.0028 (16)	0.0043 (16)
C8	0.0305 (19)	0.036 (2)	0.0309 (19)	0.0050 (15)	−0.0027 (15)	0.0032 (15)
C9	0.0340 (19)	0.035 (2)	0.0303 (19)	0.0039 (16)	0.0014 (16)	0.0063 (16)
C10	0.0294 (19)	0.040 (2)	0.0269 (18)	0.0037 (15)	−0.0052 (14)	−0.0012 (15)
C11	0.0312 (19)	0.046 (2)	0.035 (2)	0.0057 (17)	−0.0073 (16)	0.0066 (17)
C12	0.037 (2)	0.058 (3)	0.034 (2)	0.0027 (19)	−0.0115 (17)	0.0007 (19)
C13	0.043 (2)	0.051 (3)	0.037 (2)	−0.0009 (19)	−0.0102 (18)	−0.0066 (19)
C14	0.047 (2)	0.042 (2)	0.039 (2)	0.0005 (18)	−0.0119 (18)	0.0003 (18)
C15	0.0322 (19)	0.045 (2)	0.0265 (18)	0.0078 (17)	−0.0056 (15)	−0.0019 (17)
C16	0.040 (2)	0.036 (2)	0.039 (2)	0.0007 (17)	−0.0130 (17)	0.0023 (17)
C17	0.045 (2)	0.030 (2)	0.037 (2)	0.0020 (17)	−0.0077 (18)	0.0056 (17)
C18	0.052 (2)	0.048 (2)	0.043 (2)	0.006 (2)	−0.0136 (19)	0.0080 (19)
C19	0.057 (3)	0.068 (3)	0.048 (3)	0.007 (2)	−0.013 (2)	0.000 (2)

### Geometric parameters (Å, º)

**Table:**

O1—N1	1.226 (4)	C7—C8	1.396 (5)
O2—N1	1.224 (4)	C8—C9	1.399 (4)
O3—C7	1.281 (4)	C8—H8	0.9500
O3—H3A	0.8600	C9—C10	1.470 (5)
O4—C9	1.311 (4)	C10—C15	1.394 (5)
O4—H4A	0.8601	C10—C11	1.404 (4)
O5—C15	1.375 (4)	C11—C12	1.371 (5)
O5—C16	1.412 (4)	C11—H11	0.9500
O6—C17	1.189 (4)	C12—C13	1.371 (5)
O7—C17	1.327 (4)	C12—H12	0.9500
O7—C18	1.464 (4)	C13—C14	1.390 (5)
N1—C4	1.480 (4)	C13—H13	0.9500
C1—C6	1.388 (4)	C14—C15	1.387 (5)
C1—C2	1.390 (5)	C14—H14	0.9500
C1—C7	1.500 (5)	C16—C17	1.510 (5)
C2—C3	1.381 (5)	C16—H16A	0.9900
C2—H2	0.9500	C16—H16B	0.9900
C3—C4	1.374 (5)	C18—C19	1.486 (5)
C3—H3	0.9500	C18—H18A	0.9900
C4—C5	1.381 (5)	C18—H18B	0.9900
C5—C6	1.392 (5)	C19—H19A	0.9800
C5—H5	0.9500	C19—H19B	0.9800
C6—H6	0.9500	C19—H19C	0.9800
			
C7—O3—H3A	112.1	C12—C11—C10	122.0 (3)
C9—O4—H4A	103.8	C12—C11—H11	119.0
C15—O5—C16	117.3 (3)	C10—C11—H11	119.0
C17—O7—C18	118.2 (3)	C13—C12—C11	120.0 (3)
O2—N1—O1	124.2 (3)	C13—C12—H12	120.0
O2—N1—C4	118.4 (3)	C11—C12—H12	120.0
O1—N1—C4	117.4 (3)	C12—C13—C14	119.9 (3)
C6—C1—C2	119.4 (3)	C12—C13—H13	120.1
C6—C1—C7	121.6 (3)	C14—C13—H13	120.1
C2—C1—C7	118.9 (3)	C15—C14—C13	120.1 (4)
C3—C2—C1	120.6 (3)	C15—C14—H14	120.0
C3—C2—H2	119.7	C13—C14—H14	120.0
C1—C2—H2	119.7	O5—C15—C14	121.9 (3)
C4—C3—C2	118.7 (3)	O5—C15—C10	117.3 (3)
C4—C3—H3	120.6	C14—C15—C10	120.8 (3)
C2—C3—H3	120.6	O5—C16—C17	108.0 (3)
C3—C4—C5	122.5 (3)	O5—C16—H16A	110.1
C3—C4—N1	119.8 (3)	C17—C16—H16A	110.1
C5—C4—N1	117.7 (3)	O5—C16—H16B	110.1
C4—C5—C6	118.1 (3)	C17—C16—H16B	110.1
C4—C5—H5	121.0	H16A—C16—H16B	108.4
C6—C5—H5	121.0	O6—C17—O7	125.6 (3)
C1—C6—C5	120.6 (3)	O6—C17—C16	126.7 (3)
C1—C6—H6	119.7	O7—C17—C16	107.7 (3)
C5—C6—H6	119.7	O7—C18—C19	109.0 (3)
O3—C7—C8	122.5 (3)	O7—C18—H18A	109.9
O3—C7—C1	115.5 (3)	C19—C18—H18A	109.9
C8—C7—C1	122.1 (3)	O7—C18—H18B	109.9
C7—C8—C9	120.0 (3)	C19—C18—H18B	109.9
C7—C8—H8	120.0	H18A—C18—H18B	108.3
C9—C8—H8	120.0	C18—C19—H19A	109.5
O4—C9—C8	118.2 (3)	C18—C19—H19B	109.5
O4—C9—C10	115.0 (3)	H19A—C19—H19B	109.5
C8—C9—C10	126.8 (3)	C18—C19—H19C	109.5
C15—C10—C11	117.2 (3)	H19A—C19—H19C	109.5
C15—C10—C9	125.7 (3)	H19B—C19—H19C	109.5
C11—C10—C9	117.0 (3)		
			
C6—C1—C2—C3	−1.1 (5)	C8—C9—C10—C15	8.1 (6)
C7—C1—C2—C3	−179.9 (3)	O4—C9—C10—C11	7.8 (5)
C1—C2—C3—C4	1.0 (6)	C8—C9—C10—C11	−172.5 (3)
C2—C3—C4—C5	−0.2 (6)	C15—C10—C11—C12	0.0 (5)
C2—C3—C4—N1	180.0 (3)	C9—C10—C11—C12	−179.5 (3)
O2—N1—C4—C3	−177.9 (3)	C10—C11—C12—C13	0.0 (6)
O1—N1—C4—C3	2.6 (5)	C11—C12—C13—C14	0.1 (6)
O2—N1—C4—C5	2.3 (5)	C12—C13—C14—C15	−0.4 (6)
O1—N1—C4—C5	−177.2 (3)	C16—O5—C15—C14	4.2 (5)
C3—C4—C5—C6	−0.4 (6)	C16—O5—C15—C10	−177.2 (3)
N1—C4—C5—C6	179.4 (3)	C13—C14—C15—O5	178.9 (3)
C2—C1—C6—C5	0.5 (5)	C13—C14—C15—C10	0.4 (6)
C7—C1—C6—C5	179.2 (3)	C11—C10—C15—O5	−178.8 (3)
C4—C5—C6—C1	0.2 (5)	C9—C10—C15—O5	0.6 (5)
C6—C1—C7—O3	−177.2 (3)	C11—C10—C15—C14	−0.3 (5)
C2—C1—C7—O3	1.6 (5)	C9—C10—C15—C14	179.2 (3)
C6—C1—C7—C8	3.6 (5)	C15—O5—C16—C17	173.6 (3)
C2—C1—C7—C8	−177.7 (3)	C18—O7—C17—O6	2.1 (6)
O3—C7—C8—C9	−0.1 (5)	C18—O7—C17—C16	−178.9 (3)
C1—C7—C8—C9	179.1 (3)	O5—C16—C17—O6	4.0 (6)
C7—C8—C9—O4	0.5 (5)	O5—C16—C17—O7	−175.0 (3)
C7—C8—C9—C10	−179.2 (3)	C17—O7—C18—C19	97.7 (4)
O4—C9—C10—C15	−171.6 (3)		

### Hydrogen-bond geometry (Å, º)

**Table:**

*D*—H···*A*	*D*—H	H···*A*	*D*···*A*	*D*—H···*A*
C8—H8···O5	0.95	2.18	2.796 (4)	122
C16—H16*A*···O3^i^	0.99	2.35	3.295 (5)	160
O3—H3*A*···O4	0.86	1.69	2.435 (3)	144
O4—H4*A*···O3	0.86	1.62	2.435 (3)	158

Symmetry code: (i) −*x*+1/2, *y*−1/2, −*z*+1/2.
