# Supplementary figures and images for: Crystal structure of ethyl 2-{2-[(1Z)-1-hy­droxy-3-(4-nitro­phen­yl)-3-oxoprop-1-en-1-yl]phen­oxy}acetate
Source: Acta Crystallogr E Crystallogr Commun. 2015 Nov 7;71(Pt 12):o917–8. doi: 10.1107/S2056989015020794 (PMC4719879; doi:10.1107/S2056989015020794)

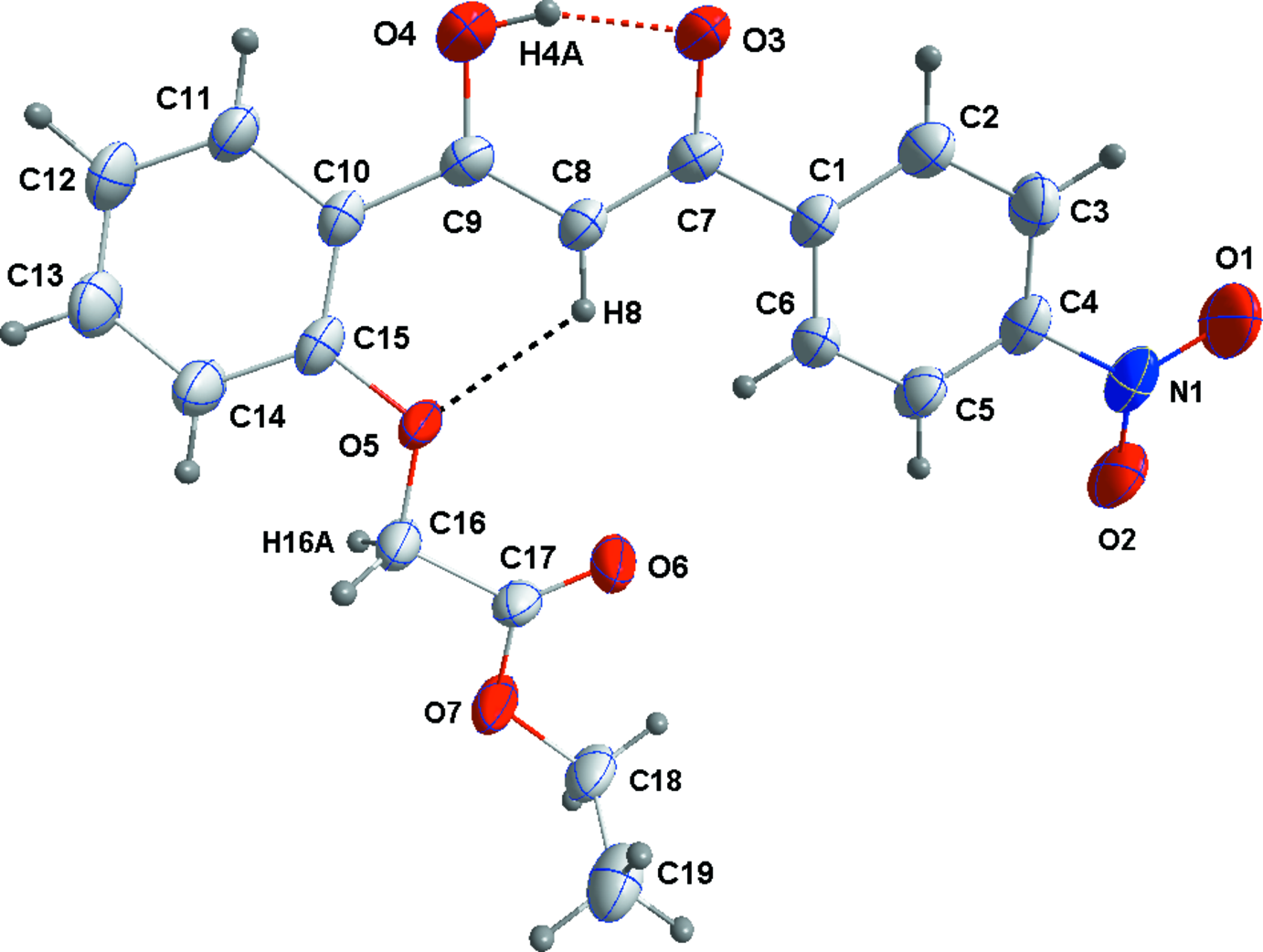

Supplement: Supplementary file 4 [file e-71-0o917-fig1.tif]

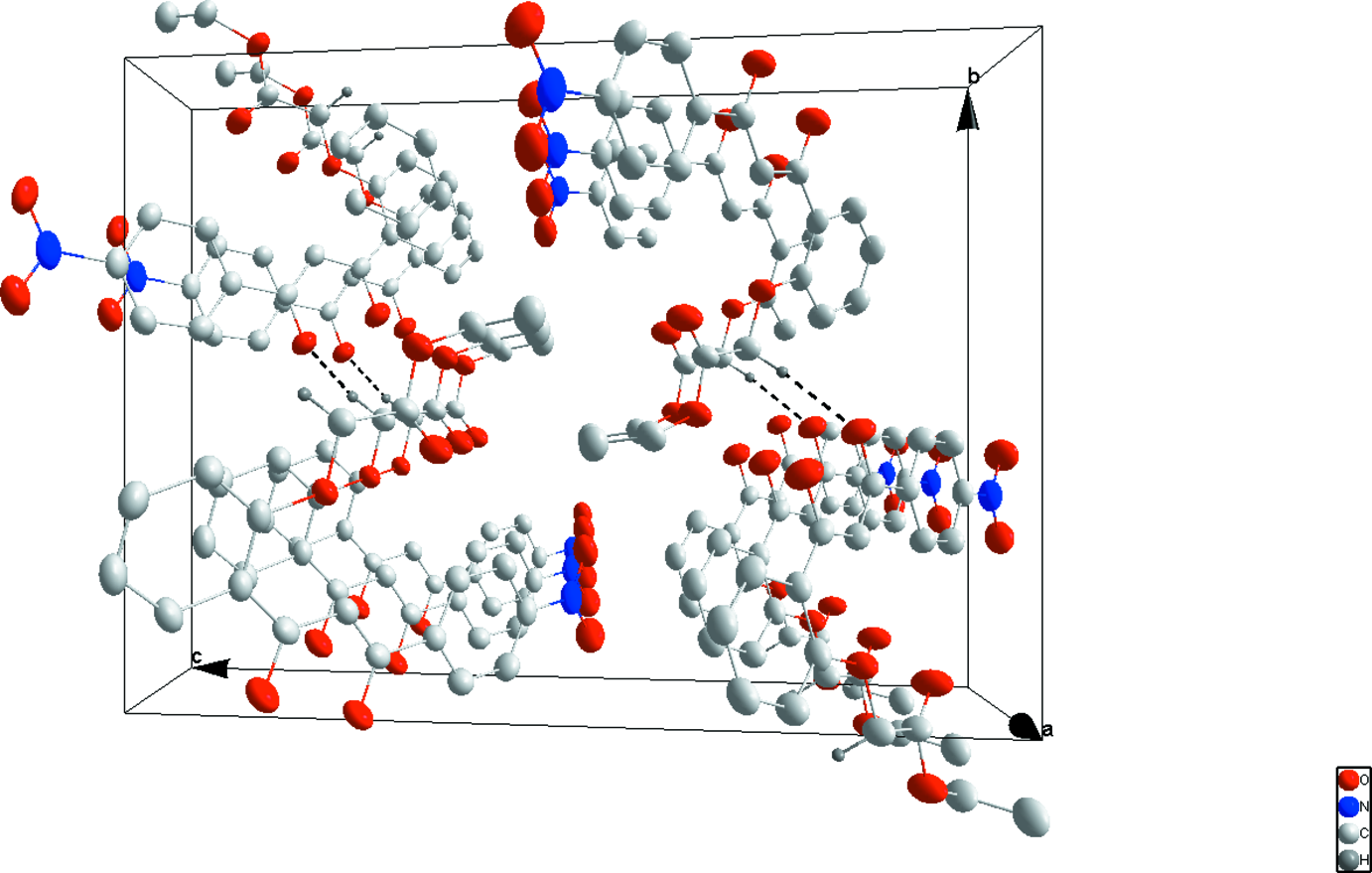

Supplement: Supplementary file 5 [file e-71-0o917-fig2.tif]

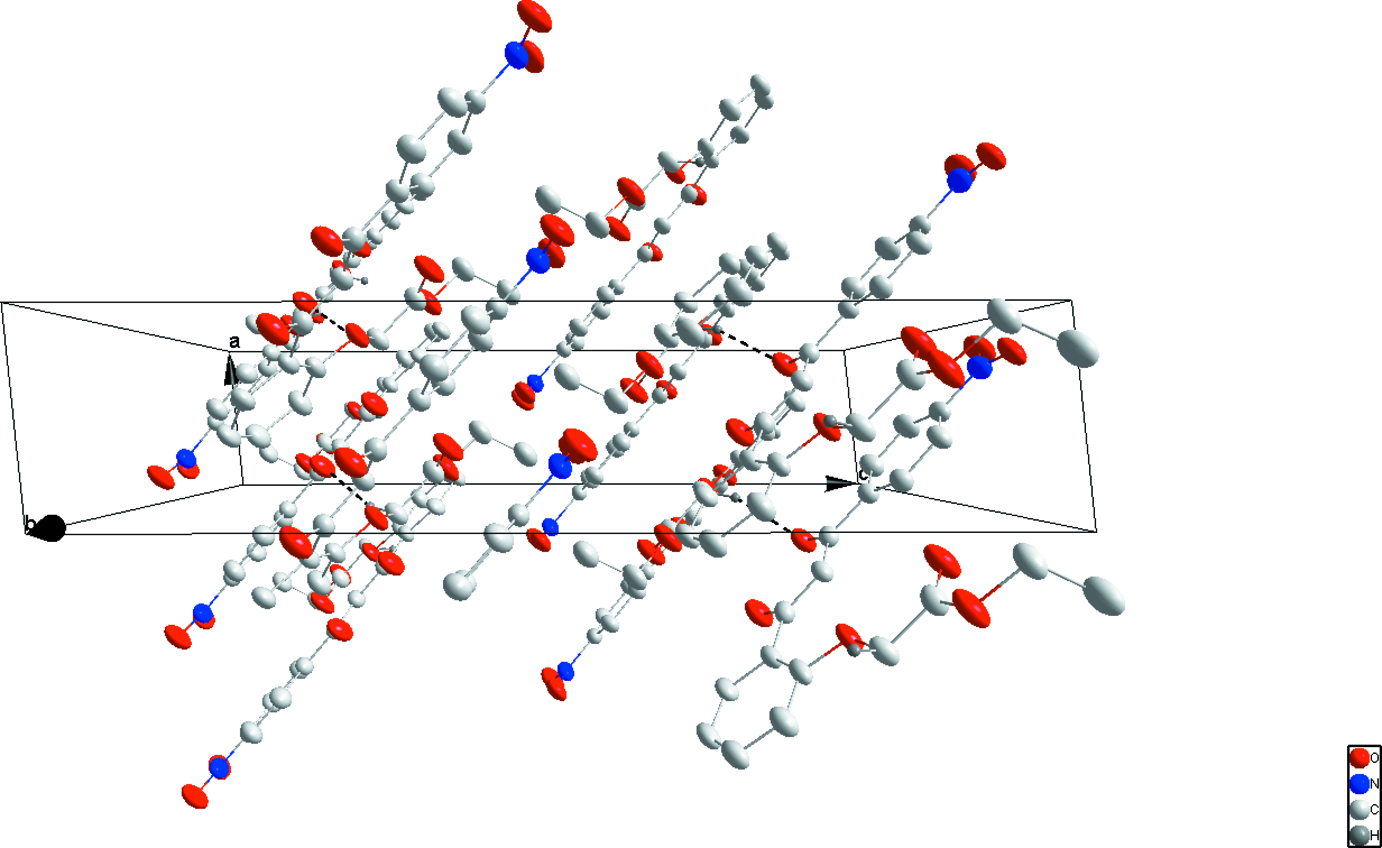

Supplement: Supplementary file 6 [file e-71-0o917-fig3.tif]
